# Estimation of the protein–ligand interaction energy for model building and validation

**DOI:** 10.1107/S2059798317003400

**Published:** 2017-03-06

**Authors:** Daria A. Beshnova, Joana Pereira, Victor S. Lamzin

**Affiliations:** a European Molecular Biology Laboratory, c/o DESY, Notkestrasse 85, 22607 Hamburg, Germany

**Keywords:** protein–ligand interactions, structure validation, automated identification of crystallographic ligands, protein–ligand interaction energy, *ARP/wARP*, *LigEnergy*

## Abstract

Macromolecular X-ray crystallography is one of the main experimental techniques to visualize protein–ligand interactions. The high complexity of the ligand universe, however, has delayed the development of efficient methods for the automated identification, fitting and validation of ligands in their electron-density clusters. The identification and fitting are primarily based on the density itself and do not take into account the protein environment, which is a step that is only taken during the validation of the proposed binding mode. Here, a new approach, based on the estimation of the major energetic terms of protein–ligand interaction, is introduced for the automated identification of crystallo­graphic ligands in the indicated binding site with *ARP*/*wARP*. The applicability of the method to the validation of protein–ligand models from the Protein Data Bank is demonstrated by the detection of models that are ‘questionable’ and the pinpointing of unfavourable interatomic contacts.

## Abstract 2

The use of the protein–ligand interaction energy as an additional parameter for the automated identification of crystallographic ligands and its applicability to structure validation are described.

## 1. Introduction   

The understanding of biochemical processes relies on the derived knowledge on how macromolecules, in their biological context, interact with a wide range of small molecules: the ligands. A number of tools exist that provide a means to study these interactions, and macromolecular X-ray crystallography (MX) is used as the main experimental technique for structural analysis. While the model-building procedure for proteins depends on the known sequence of the macromolecule and the quality of the data, ligand identification and fitting presents a number of challenges. Firstly, the universe of small molecules that interact with proteins is vast, and the ligands may feature different complexities, shapes and topologies (Stockwell & Thornton, 2006 ▸). Indeed, the Protein Data Bank (PDB; Berman *et al.*, 2000 ▸) contains more than 20 000 distinct ligands or small molecules bound to proteins and nucleic acids (Velankar *et al.*, 2010 ▸; Sen *et al.*, 2014 ▸). Secondly, ligands can be partially disordered owing to an insufficiently high binding affinity or conformational flexibility, and this may present a difficulty in their identification and modelling (Liebeschuetz *et al.*, 2012 ▸; Pozharski *et al.*, 2013 ▸). Thirdly, there are cases in which several ligands bind to the same binding site at the same time, and this results in partially occupied overlapping networks (Ma *et al.*, 2002 ▸). Therefore, approaches for modelling ligands have always been in a less advanced state than those for proteins.

The increased interest in structure-based drug design has promoted the development of methods for the automated building of small molecules in electron-density maps and their software implementation. *PHENIX* (Adams *et al.*, 2010 ▸), *Coot* (Emsley & Cowtan, 2004 ▸; Debreczeni & Emsley, 2012 ▸) and *ARP*/*wARP* (Langer *et al.*, 2008 ▸) are examples of academic software packages that are widely used for crystallographic ligand building. In addition, the *Privateer* software (Agirre *et al.*, 2015 ▸) has been specifically designed for the modelling and validation of carbohydrates. All of these packages apply different methods and approaches to accomplish the same task: to maximize the fit of the ligand to the experimentally derived electron density. For example, *ARP*/*wARP* methods are based on the identification of atomic features in the identified density cluster and their further interpretation in terms of connectivity and conformation (Langer *et al.*, 2012 ▸). The *PHENIX* method searches for the location of rigid parts of the ligand and then accomplishes their extension by following the density shape (Terwilliger *et al.*, 2006 ▸). The *Coot* package proceeds by identifying the density that fits pre­defined conformations of the ligand and then adjusts the most suitable ligand model through its real-space fit to the density (Emsley & Cowtan, 2004 ▸).

These methods also provide tools for the identification of possible binding sites in cases where the search ligand is known but the corresponding density cluster is not. In addition, it may be possible to guess the ligand identity from the defined density cluster. For example, in ligand guessing and identification of the binding site in *ARP*/*wARP*, a major role is played by analysis of the density shape; a number of numerical descriptors are calculated for a set of most common ligands in different conformations (Carolan & Lamzin, 2014 ▸), while a search of the density cluster is accomplished using the so-called fragmentation tree (Langer *et al.*, 2012 ▸). In *PHENIX*, all possible binding sites for a set of ligands are identified by a search for contiguous regions of density and the identity of the most likely ligand is guessed using the density fit; the ligand selected is that which has the best real-space correlation and surface complementarity to the protein atoms surrounding the binding site (Terwilliger *et al.*, 2007 ▸; Adams *et al.*, 2010 ▸). An option to screen a cocktail of possible ligands is provided in *Coot* (Debreczeni & Emsley, 2012 ▸) and *ARP*/*wARP* (Langer *et al.*, 2008 ▸).

Although differing in detail, all of these methods have in common the maximization of a scoring function, which is mostly dependent on the geometry and conformation of the ligand and its fit to the density. Some interactions arising from the binding mode are considered during the validation of an already built ligand but not during the model-building process. For example, *phenix.ligand_identification* checks the surface complementarity with the atoms surrounding the binding site after the identification of an unknown ligand in a density cluster (Adams *et al.*, 2010 ▸). *ARP*/*wARP* warns if the built ligand has steric clashes within itself. *WHAT_CHECK* (Hooft *et al.*, 1996 ▸), *WHAT_IF* (Rodriguez *et al.*, 1998 ▸) and *MolProbity* (Chen *et al.*, 2010 ▸) can identify the presence of atomic clashes in the structure. Additionally, *MolProbity* uses the small-probe contact dot surface analysis to visualize van der Waals (VDW) and hydrogen-bond contacts and atomic clashes. This option is also implemented in *Coot* (Debreczeni & Emsley, 2012 ▸).

The estimation of the energetics of ligand-binding modes has been a general tool for ligand scoring in structure-based drug-design and screening projects for a decade (Kitchen *et al.*, 2004 ▸). However, as yet no academic crystallographic package includes such a scoring function during ligand guessing, building or validation. One reason is related to the fact that a truly accurate estimation of the energy term is difficult to achieve.

Here, we propose a novel approach, *LigEnergy*, for the evaluation of protein–ligand binding in MX, which is based on estimation of the protein–ligand interaction energy (Pacholczyk & Kimmel, 2011 ▸). The estimation is obtained using a simplification of the semi-empirical force field as implemented in *AutoDock* for the docking of the ligand to a target protein (Huey *et al.*, 2007 ▸; Morris *et al.*, 2009 ▸). This energy term, normalized by the number of non-H ligand atoms, offers a single-parameter estimator of the quality of a modelled protein–ligand complex. The method allows the fast scanning of large databases and the identification of protein–ligand complexes which are ‘questionable’. At the same time, the method allows an improvement of the identification and fitting of ligands into specified electron density with *ARP*/*wARP*.

## 2. Theory and methods   

### 2.1. Test cases   

In order to develop and test *LigEnergy* for its application to the validation of protein–ligand complexes, a set of structural entries were collected from the PDB as follows. Firstly, filtering using the PDB Advanced Search tools was performed. The entries were selected provided that the models contained ligands and protein components, were obtained by X-ray crystallography at a resolution of 3.0 Å or better, and contained the experimental data. Secondly, only representatives at 50% sequence identity were considered, resulting in a set of 17 523 PDB entries. Thirdly, for each model only the largest noncovalently bound ligand molecule with 10–50 non-H atoms was considered. If several copies of the same ligand or several protein chains were present (for example, in homo-multimers), only the ligand corresponding to the first protein chain was investigated. Overall, 4771 protein–ligand complexes, comprising 1228 unique noncovalently bound ligands, were collected.

The average atomic displacement parameter (ADP) and atomic occupancy were computed for each ligand, and the real-space correlation coefficient (RSCC) to the experimental data was used as provided by the Uppsala Electron Density Server (EDS; Kleywegt *et al.*, 2004 ▸). The distributions of the RSCC, the average occupancy of the ligand atoms and the average ADP were then inspected (Supplementary Fig. S1). The entries were kept if the ligand molecule was fully occupied and had an average ADP below 80 Å^2^ and an RSCC higher than 0.917 (the top 50% of cases). As a result, a total of 2020 protein–ligand complexes, comprising 660 unique ligands, were used in further studies.

To test the utility of *LigEnergy* for the automated identification of crystallographic ligands (ligand guessing), from these 2020 protein–ligand complexes we selected 100 structures containing ligands that are present in the *ARP*/*wARP* ligand-guessing database (Carolan & Lamzin, 2014 ▸).

### 2.2. Estimation of the protein–ligand interaction energy   

The value of the free energy of binding can be used for the scoring of protein–ligand complexes (Kitchen *et al.*, 2004 ▸). While several classes of scoring functions exist, semi-empirical free-energy force-field functions provide a fast tool for the estimation of the free energy of binding (Huey *et al.*, 2007 ▸). Several force-field approaches exist for a description of biochemical systems, with the most common being AMBER (Weiner & Kollman, 1981 ▸; Cornell *et al.*, 1995 ▸; Duan *et al.*, 2003 ▸; Hornak *et al.*, 2006 ▸), CHARMM (Brooks *et al.*, 1983 ▸), GROMOS (Scott *et al.*, 1999 ▸) and OPLS (Jorgensen *et al.*, 1996 ▸). All these approaches use similar VDW and Coulomb potentials for estimation of the interaction energy of non­bonded atoms.

In macromolecular crystallographic ligand building or validation, however, the interest is not necessarily in the absolute value of the free energy of ligand binding to the protein. Specifically, here we are interested in testing a hypothesis as to whether an observed or proposed bound state is feasible from an energetic point of view. For this, only the energy of the protein–ligand complex in the bound state is required.

For this, we present here a protein–ligand bound-state energy estimator using the semi-empirical force field as approximated and implemented in *AutoDock* 4 for ligand scoring (Huey *et al.*, 2007 ▸; Morris *et al.*, 2009 ▸). The *AutoDock* free-energy scoring function is based on the AMBER force field and was parameterized using a large number of protein–inhibitor complexes for which both the structure and the inhibition constants were known (Morris *et al.*, 2009 ▸). Here, we use a function for the energy estimation as described in Huey *et al.* (2007 ▸) and Morris *et al.* (1998 ▸) and compute the interaction energy of the protein–ligand bound state (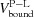), 
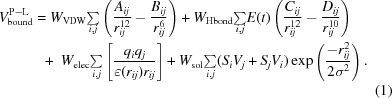
The first term corresponds to the 6–12 Lennard–Jones potential for the dispersion/repulsion interactions, with parameters *A* and *B* taken from the AMBER force field (Weiner *et al.*, 1984 ▸). The second term presents a hydrogen-bond energy estimated by a 10–12 potential (Morris *et al.*, 1998 ▸). The third term is the energy of electrostatic interactions, based on the Coulomb potential, with the distance-dependent dielectric constant ∊(*r*_*ij*_) (Mehler & Solmajer, 1991 ▸). The parameters *C* and *D*, the directionality of the hydrogen-bond interaction *E*(*t*), which depends on the deviation of the angle *t* from ideal bonding geometry, and the optimized weights *W*_vdw_, *W*_Hbond_, *W*_elec_ and *W*_sol_ were calculated according to Boobbyer *et al.* (1989 ▸) and Huey *et al.* (2007 ▸). The last term is a desolvation potential as described in Huey *et al.* (2007 ▸). This term includes the volume (*V*) surrounding a given atom, weighted by the solvation parameter (*S*) and an exponential term based on the distance.

For the assignment of partial charges to the ligand and protein atoms, the Marsili–Gasteiger partial charges were calculated on the basis of electronegativity equilibration using the ‘partial equalization of orbital electronegativities’ method (Hinze & Jaffe, 1962 ▸; Hinze *et al.*, 1963 ▸; Gasteiger & Marsili, 1980 ▸). For hydrogen bonding we consider here only polar H atoms calculated at their riding position. Both the partial charges and the locations of polar H atoms were computed using *AutoDockTools* (Sanner, 1999 ▸; Morris *et al.*, 2009 ▸).

### 2.3. *LigEnergy* for the validation of bound ligands   

**Figure 1:**
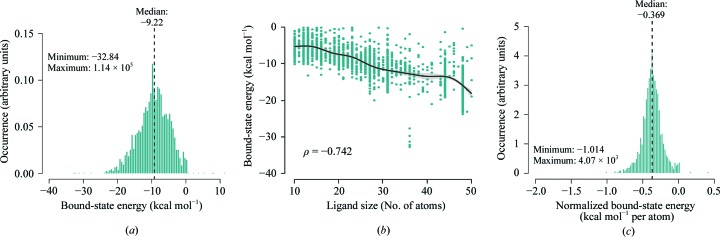
The energy of the bound state for the selected protein–ligand complexes. (*a*) The distribution of the energy computed; outliers with highly positive values are not shown. The median, minimum and maximum values are indicated. (*b*) The energy of the bound state as a function of the number of non-H ligand atoms. The Pearson linear correlation coefficient (ρ) and the smooth conditional mean (computed with *ggplot*2; Wickham, 2009 ▸) are shown. (*c*) Distribution of the bound-state energy normalized by the number of non-H ligand atoms; outliers with highly positive values are not shown. The median, minimum and maximum values are indicated.

The energy of the protein–ligand bound state, estimated using (1), shows a linear dependence on the number of non-H ligand atoms (Fig. 1 ▸ *b*). Normalization of (1) by the number of ligand atoms results in an energetic metric (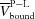) that has a bell-shaped distribution (Fig. 1 ▸ *c*) and corresponds to the mean energy contribution of each atom in the ligand when it is bound to the protein (hereafter denoted the *normalized energy*).

In MX the RSCC value is used for estimation of the overall fit of the model to the electron density. For the validation of deposited protein–ligand structures, in addition to the normalized energy, we also use information about the density support. If a ligand has high-density support but unfavourable interactions, it may either require further refinement or be incorrect. If no density support exists, even if the energy of the proposed bound state is favourable, then the proposed binding mode is regarded as ‘questionable’.

### 2.4. *LigEnergy* for the guessing of bound ligands   

We used the ligand-guessing method as implemented in *ARP*/*wARP* v.7.6 to identify 40 top candidate compounds by the comparison of the shape descriptors of ligands from the database and that of the binding site, complemented by their RSCC. The *LigEnergy* approach was subsequently used to assist in the ranking of these 40 compounds by taking into account the protein–ligand interaction energies. As described below, we found that the combination of a density-fit term (RSCC) with the normalized energy term (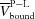) is preferred compared with the use of any of these terms alone. Therefore, the *LigEnergy* scoring function for ligand guessing is computed as a nonparametric average of the two terms, 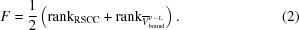



## 3. Results   

### 3.1. Energy distribution of the deposited protein–ligand complexes   

For the selected set of 2020 deposited protein–ligand models with an RSCC for the ligand above 0.917, the distribution of the computed bound-state energy has a bell-shaped distribution with a median value of −9.22 kcal mol^−1^ (Fig. 1 ▸ *a*). While the minimum value of the energy is about −33 kcal mol^−1^, there are 22 cases with a positive energy value. Without these, there is a linear correlation of the energy of the bound state with the number of ligand non-H atoms (Fig. 1 ▸ *b*), with a Pearson correlation coefficient of −0.74. Normalization by the number of non-H atoms decorrelates the energy of the bound state from the ligand size and results in the normalized energy having a more symmetric bell-shaped distribution (Fig. 1 ▸ *c*). The median normalized energy is −0.37 kcal mol^−1^ per atom. A closer inspection of the cases showed that those with a positive energy contain interatomic clashes. Ligand models with a value of the normalized energy of the bound state between 0.0 and −0.1 kcal mol^−1^ per atom typically form very weak contacts with the protein, and ligand binding occurs at the protein surface. Finally, the models with normalized energy values below −0.1 kcal mol^−1^ per atom do not display any problems in the protein–ligand interface. These results additionally confirm that the value of the RSCC alone may not be sufficient to classify a ligand as well modelled. In the next section some examples will be described in more detail.

### 3.2. Validation of the deposited models   

The values of the interaction energy calculated for the whole data set are given in Supplementary Table S1. Five cases out of the selected 2020 stood out owing to their extremely low energy: −32 kcal mol^−1^ (Fig. 1 ▸ *c*). These correspond to the same ligand: inositol hexakisphosphate (IHP) or phytic acid (PDB entries 5hdt, 3ho6, 1zy7, 3pev and 2p1m). This ligand has 36 non-H atoms and is an important signalling molecule that influences the permeability of ion channels, the regulation of transcriptional response to environmental arginine in yeast, insulin secretion from pancreatic β cells, embryotic development *etc.* (reviewed by Hatch & York, 2010 ▸). It has a normalized energy of −0.9 kcal mol^−1^ per atom as a result of a high number of favourable electrostatic interactions between its six negatively charged phosphate groups and a positively charged protein-binding pocket (Pruitt *et al.*, 2009 ▸).

The majority of the selected protein–ligand models (1925 structures or 95%) show a negative normalized energy below −0.1 kcal mol^−1^ per atom. There are 95 complexes that have normalized energies between −0.1 and 0 kcal mol^−1^ per atom. These may contain an interatomic clash, have weak binding or correspond to a nonspecific protein–ligand interaction (Lepre *et al.*, 2004 ▸). Indeed, it is known that substances added for protein crystallization such as buffers, cryoprotectants or polymers may bind to the protein surface with possibly little relevance to protein function.

**Figure 2:**
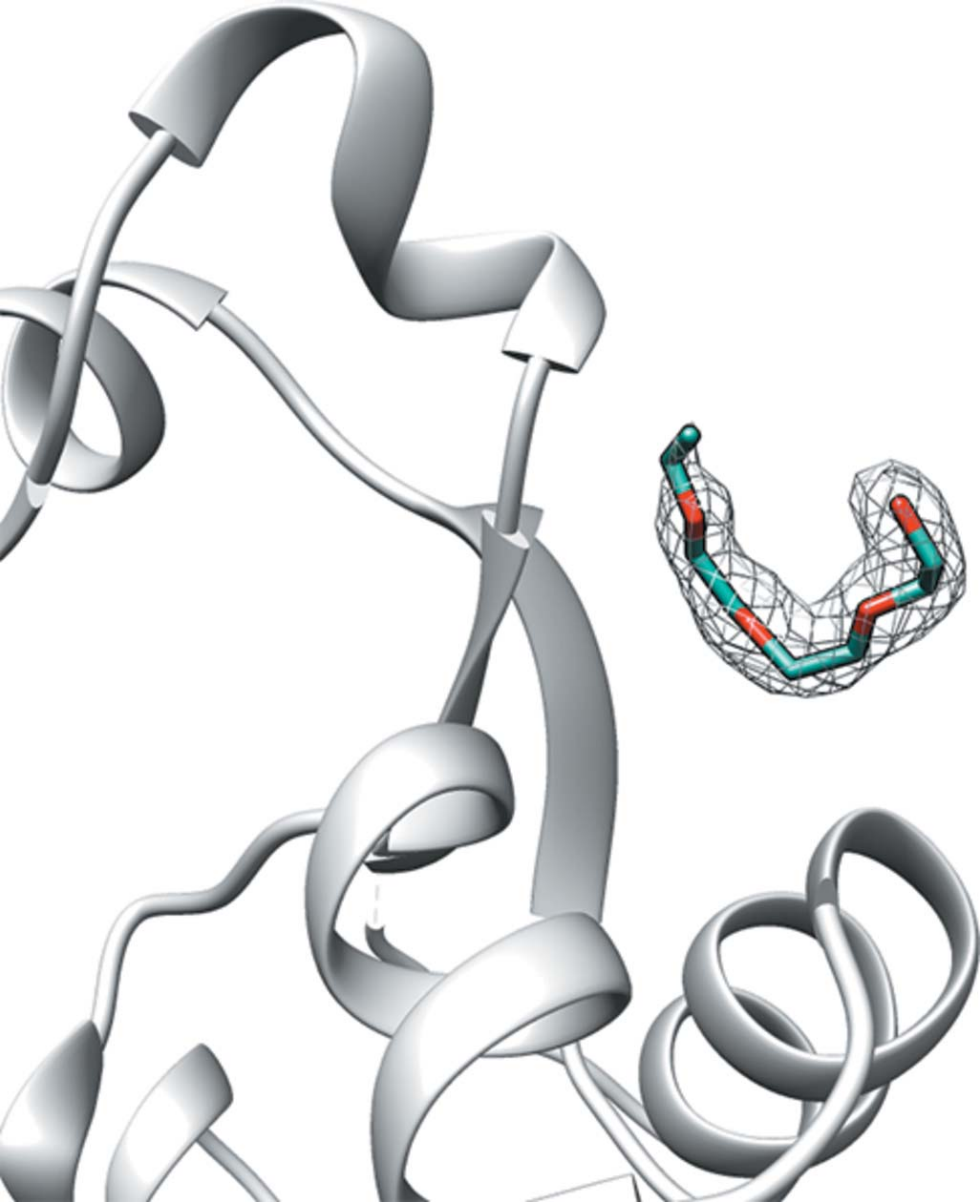
An example of weak protein–ligand interaction: a tandem winged-helix domain of RNA polymerase I subunit A49 in complex with a fragment of polyethylene glycol PEG 4000 (PE4).

In 29 of the above mentioned 95 complexes, we detected no or very weak VDW, electrostatic and/or hydrogen-bond interactions; therefore, their normalized protein–ligand energy is equal to 0 kcal mol^−1^. These 29 complexes contain the polymers hexaethylene glycol (PG4), pentaethylene glycol (1PE) and a fragment of polyethylene glycol PEG 400 (PE4), triethylene glycol (PGE), the buffers CASP, MES, EPE and CIT, *etc.* An example of such weak interaction with a polymer molecule used during crystallization is presented in Fig. 2 ▸ (PDB entry 3nfi).

22 test cases show a positive normalized energy value. Their detailed inspection revealed the presence of a number of severe clashes between the protein and ligand atoms. One example is the complex between guanosine 5′-diphosphate and full-length *Thermus thermophilus* apo IF2 (PDB entry 4kjz), which has the highest normalized energy among all analysed structures: 4071 kcal mol^−1^ per atom. There are very close contacts between the atoms Lys181A NZ and the ligand O4′ (1.0 Å) and C1′ (1.3 Å) atoms, between Val82A O and the ligand O1B atom (1.6 Å) *etc.* The estimated *MolProbity* score for this structure is also high, 250. We note that some structures (for example, the protein–ligand complex with PDB code 3ihj with a normalized protein–ligand interaction energy of 1500 kcal mol^−1^) contain interatomic contacts that are seemingly too short. However, detailed inspection revealed that in the case of PDB entry 3ihj these contacts are indeed the covalent bonds, which are not clearly annotated in the PDB file.

### 3.3. Automated identification of crystallographic ligands assisted by the use of *LigEnergy*   

We examined the use of the protein–ligand interaction energy as an additional parameter for the improvement of ligand-guessing protocols during the automated identification of ligands using sparse-density representations with *ARP*/*wARP* (Carolan & Lamzin, 2014 ▸). The method compares the shape descriptors of the ligands from a database with those of the binding site and uses the values of RSCC for the selected ligands as the scoring function. While the method generally works well, mistakes in pointing to the correct ligand for a given binding site do appear, particularly when the electron density is poor. We suggest that for the final stages of ligand ranking additional parameters would help to identify the most likely binding partner with a higher degree of confidence. Here, we considered 100 cases from the selected set of protein–ligand complexes, with the aim of examining whether the *LigEnergy* approach would help in guessing the deposited ligand (Supplementary Table S2). In 50 cases the ligand-guessing method gave an adequate grid representation of the density and for 32 structures it correctly identified the ligand in the first place in the ranking. With the help of *LigEnergy* (2), all cases with the adequate grid were built correctly (50) and the correct ligand was always identified as the top one (Supplementary Table S2). Below, we present three of these cases where *LigEnergy* helps to identify the deposited ligand in more detail.

**Figure 3:**
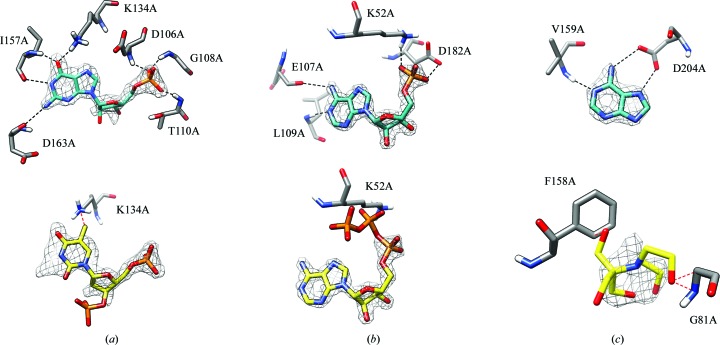
Ligand guessing without (yellow skeleton) and with (blue skeleton) the use of the estimated energy as an additional parameter for (*a*) the hypoxanthine-guanine-xanthine phosphoribosyltransferase [PDB entry 1hgx; 2*mF*_o_ − *mF*_c_ map contoured at a 2.5σ level above the mean (0.833 e Å^−3^) in black mesh], (*b*) the Bud32–Cgi121 protein complex [PDB entry 4ww7; 2*mF*_o_ − *mF*_c_ map contoured at a 2.5σ level above the mean (0.709 e Å^−3^) in black mesh] and (*c*) the putative 5′-methylthioadenosine/*S*-adenosylhomocysteine nucleosidase from *B. burgdorferi* B31 [PDB entry 4l0m; 2*mF*_o_ − *mF*_c_ map contoured at a 2.5σ level above the mean (0.831 e Å^−3^) in black mesh]. Dashed black lines indicate favourable contacts; dashed red lines show interatomic clashes.

The first case comprises the guanosine 5′-monophosphate (5GP) ligand bound to the hypoxanthine-guanine-xanthine phosphoribosyltransferase (PDB entry 1hgx; Somoza *et al.*, 1996 ▸). The existing ligand-guessing protocol identified a thymidine 3′,5′-diphosphate (THP) molecule as the most likely binder in the electron-density cluster present at the binding site. However, the ligand with the best interaction energy (−0.32 kcal mol^−1^ per atom) was indeed 5GP, the deposited ligand, which forms favourable hydrogen bonds with protein chains (Fig. 3 ▸ *a* and Supplementary Table S2). The incorrectly identified THP ligand forms a clash between its C atom and the Lys134A N atom.

Another case is the complex between adenosine monophosphate (AMP) and the binary complex of the Bud32 and Cgi121 proteins (PDB entry 4ww7; Zhang *et al.*, 2015 ▸). The ligand-guessing protocol identified adenosine triphosphate (ATP) as the most likely binder for this complex (Fig. 3 ▸ *b*). However, a closer inspection of the built model shows that ATP has a poor electron-density fit for its two phosphate groups, resulting in contacts with the protein Lys52 residue that are too short. Using *LigEnergy* (2), ATP was no longer at the top of the ranking (Supplementary Table S2), while AMP was instead identified as the best ligand, with a good value for the normalized energy (−0.28 kcal mol^−1^ per atom). The complex between AMP and the Bud32 and Cgi121 proteins is additionally stabilized by four hydrogen bonds between ligand atoms and Leu109, Glu107, Lys52 and Asp182 (Fig. 3 ▸ *b*).

Finally, the third test case is guessing the ligand in the difference density of the complex between an adenine (ADE) molecule and a putative 5′-methylthioadenosine/*S*-adenosyl­homocysteine nucleosidase from *Borrelia burgdorferi* B31 (PDB entry 4l0m). The existing ligand-guessing protocol suggested 2-[bis(2-hydroxyethyl)amino]-2-(hydroxymethyl)­propane-1,3-diol ligand (BTB) as the compound with the highest shape similarity to the selected protein binding site and the highest RSCC (Fig. 3 ▸ *c*). Using the *LigEnergy* approach, ADE, the correct ligand, was instead identified as the most likely binder (Fig. 3 ▸ *c*), with a normalized energy of −0.44 kcal mol^−1^ per atom. The ADE ligand forms three hydrogen bonds: between the N1 atom of the ligand and the main-chain atom of Val159, between the ligand N6 atom and Asp204 OD2, and between the ligand N7 atom and Asp204 OD1, while the modelled BTB ligand has clashes with the aromatic ring of the Phe158 side-chain atoms and the Gly81 main-chain atoms (Fig. 3 ▸ *c*).

The presented examples demonstrate that the estimated protein–ligand interaction energy can indeed serve as an additional scoring parameter for the identification of the most likely ligand for a selected density region in a given protein structure.

## 4. Discussion and conclusions   

We have presented a novel approach, *LigEnergy*, which can be used as an additional tool for evaluation of the quality of built protein–ligand complexes and for the validation of deposited models. Among 2020 selected protein–ligand complexes, we classified 95 structures (less than 5%) as ‘questionable’. Of these, 22 structures have highly positive energy resulting from severe atomic clashes between ligand and protein atoms. The other 73 complexes (3.6% of the total) have only a marginally negative energy caused by occasional clashes or complexes with nonspecific weak ligand binding. For such structures a more thorough examination of ligand binding may be advised, as it may suggest an improvement of the model. The presence of a problematic region in a protein–ligand complex may potentially lead to an incorrect interpretation of protein–ligand interactions, and this in turn may have impact on the field of drug discovery and drug design when the determined structures are used as templates (Davis *et al.*, 2008 ▸). Some existing approaches (for example, Word *et al.*, 1999 ▸; Chen *et al.*, 2010 ▸) that allow the visualization of protein–ligand contacts may not provide a sufficiently quantitative evaluation.

*LigEnergy* uses a normalized protein–ligand interaction energy, and such normalization in essence decorrelates the estimated energy and the ligand size, thus extending their applicability and interpretation for the validation of protein–ligand complexes. The more negative the normalized energy is, the more efficient the protein–ligand interaction is. This way, the normalized energy may be seen to be analogous to the ligand efficiency: the ratio between the free energy of binding (including its entropic term) and the number of non-H ligand atoms. Indeed, it has been shown that the free energy of binding correlates with the number of non-H atoms in the ligand (Kuntz *et al.*, 1999 ▸). In the same work, it was suggested that the average energy per non-H atom can be used to help find the maximum binding affinity of ligands. The ligand efficiency has been used in drug discovery to assist the identification of the optimal combination of physicochemical and pharmacological properties (Hopkins *et al.*, 2004 ▸, 2014 ▸). This metric is a measure of how well the ligand uses its atoms to interact with its targets, and allows the comparison of different ligands corrected for their size.

*LigEnergy* uses the interaction energy to offer a single-parameter estimate of the quality of protein–ligand models that may allow the fast scanning of large databases and may point to ‘questionable’ structures. The estimation of the protein–ligand interaction energy can be efficiently applied to the assessment of proposed protein–ligand models in addition to the RSCC and other quality indicators. The proposed measure is highly informative, and its value may provide a simple and rapid means of evaluating protein–ligand inter­actions and designating them as favourable or unfavourable. At the same time, detailed examination of all pairwise atomic interactions may indicate potentially problematic regions.

The presented method uses a numerical approximation, as implemented in Morris *et al.* (2009 ▸), to estimate the VDW, hydrogen-bond, electrostatic and desolvation energy terms of protein–ligand interactions. The interaction energy is strongly dependent on the interatomic distances and changes sharply at short distance values. At the same time, the models are derived from experimental techniques such as crystallography, NMR or electron microscopy, with a certain coordinate error, even in the presence of distance restraints. Such coordinate error is dependent on the quality and the amount of experimental data, but is not uniformly distributed throughout the model. It additionally depends on the local density and ligand occupancy. Use of the coordinate error for a more accurate estimation of the interaction energy could be a possible direction for future research. We also note that the *LigEnergy* approach in its present state does not take into account protein–ligand interactions through crystallographic water molecules or interactions of a ligand with another ligand or an ion. The consideration of such through-water or through-ion interactions could be another advance in the future.

The *LigEnergy* method has the potential to improve the ligand-guessing protocol during the automated identification of ligands. For the presented test cases we found that taking into account the energy of protein–ligand interaction is appropriate in a combined nonparametric use with other measures such as the RSCC. At the same time, improvement of the ligand-guessing procedure in a sparse-grid interpretation of electron density is a separate task for further research.

This study also highlights an important concern within the structural biology community that more attention should be devoted to the analysis of protein–ligand contacts and the interpretation of electron density before the structural model is submitted to the PDB (Adams *et al.*, 2016 ▸). The *LigEnergy* approach, in which we intend to provide the assignment of partial charges and the addition of H atoms using local geometry (Word *et al.*, 1999 ▸), will be implemented in *ARP*/*wARP* and will become available to the community.

## Supplementary Material

Supporting Information: Supplementary Figure S1, caption to Supplementary Table S1 and Supplementary Table S2.. DOI: 10.1107/S2059798317003400/ba5256sup1.pdf

Supporting Information: Supplementary Table S1.. DOI: 10.1107/S2059798317003400/ba5256sup2.xlsx
