# Supplementary material for: Estimation of the protein–ligand interaction energy for model building and validation
Source: Acta Crystallogr D Struct Biol. 2017 Mar 6;73(Pt 3):195–202. doi: 10.1107/S2059798317003400 (PMC5349431; doi:10.1107/S2059798317003400)
Supplement: Supplementary file 1 [file d-73-00195-sup1.pdf]

STRUCTURAL BIOLOGY

Volume 73 (2017)

Supporting information for article:

Estimation of the protein–ligand interaction energy for model building and validation

Daria Beshnova, Joana Pereira and Victor Lamzin

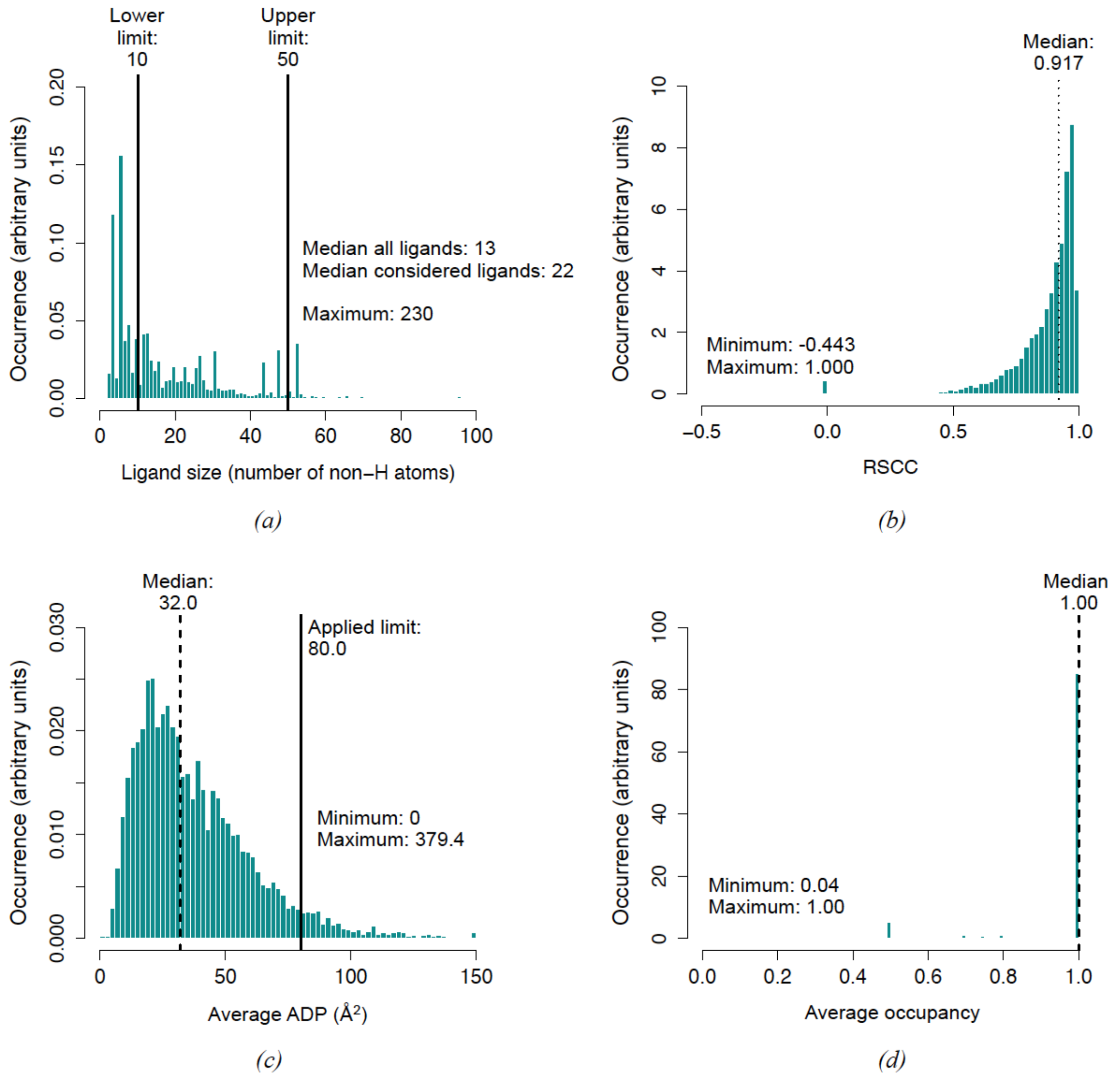

Figure S1 Distributions of (a) ligand sizes (the number of non-hydrogen atoms), (b) real space correlation coefficients (RSCC), (c) average atomic displacement parameters and (d) average atomic occupancies for the set of 9956 non-covalent protein-ligand complexes collected from the PDB. The median, minimum and maximum values are shown. When the median was not used as the threshold for filtering, the applied limit is indicated. For (a) and (c), only those cases below the applied limit were accepted, while for (b) and (d), those above the median (inclusive) were accepted.

Table S1 The values of interaction energy for 2,020 ligand-protein structures estimated by LigEnergy approach using equation (1). (See separate Excel file).

Table S2 The results of ligand identification in the electron density for 100 test cases with the help of ARP/wARP shape similarity procedure and proposed here the LigEnergy approach.

PDB ID	Ligand deposited in the PDB	Ligand identified (3-letter ligand identifier) by shape similarity and RSCC	Ligand identified (3-letter ligand identifier) by LigEnergy approach (eq. 2)
1y89	2PE	NAD	2PE
2yhw	2PE	2PE	2PE
3oa5	2PE	P6G	P6G
3vz0	2PE	PYR	PEG
1ex7	5GP	ADP	5GP
1hgx	5GP	THP	5GP
2qor	5GP	A3P	5GP
2xbu	5GP	5GP	5GP
3n1s	5GP	ATP	5GP
3tr0	5GP	ADP	5GP
1c9k	5GP	A3P	AMP
1t8t	A3P	A3P	A3P
3uan	A3P	A3P	A3P
1q1q	A3P	COA	1PE
2oq2	A3P	LDA	ORO
3ap1	A3P	COA	THP
4l0m	ADE	BTB	ADE
2pgf	ADN	NAD	ADN
3oi8	ADN	ADN	ADN
2q6k	ADN	SAM	NHE
2zbv	ADN	AMP	AMP
3o1c	ADN	SAM	NHE
1ao0	ADP	SAM	ADP
2c2a	ADP	ATP	ADP
2uyt	ADP	ADP	ADP
4rz3	ADP	ADP	ADP
1cza	ADP	SAM	COA
1in4	ADP	TPP	TYD
1um8	ADP	SAM	CMP
2w58	ADP	NAD	ATP
2yx6	ADP	H4B	AMP
3b1n	ADP	ATP	TYD
3bk7	ADP	AMP	OLA

3c4n	ADP	IPH	IPH
3cwq	ADP	TYD	TYD
2cfm	AMP	AMP	AMP
2r85	AMP	TYD	AMP
2v8q	AMP	ATP	AMP
3ber	AMP	5GP	AMP
3nzt	AMP	ATP	AMP
3o0m	AMP	ADP	AMP
4eei	AMP	AMP	AMP
4eql	AMP	AMP	AMP
4ts5	AMP	NAD	AMP
4ww7	AMP	ATP	AMP
1ry2	AMP	017	PLP
1zn8	AMP	ATP	5GP
2c5s	AMP	C2E	NHE
2gxq	AMP	ORO	ORO
2i4i	AMP	MLT	MLT
2qga	AMP	TYD	TYD
3ddj	AMP	5GP	5GP
3fwz	AMP	ADP	ADP
3glv	AMP	TPP	TPP
1jjv	ATP	ATP	ATP
2e5y	ATP	ATP	ATP
3a8t	ATP	NAP	ATP
3lgx	ATP	ATP	ATP
3rk1	ATP	ATP	ATP
2f02	ATP	FPP	FPP
2fgh	ATP	NAD	NAD
2pze	ATP	NAP	NAP
3h1q	ATP	ADP	PYR
3h39	ATP	BCL	MYR
3ikh	ATP	NAD	1PE
3lkk	ATP	LDA	CIT
3ll3	ATP	TYD	TYD
2ooq	B3P	B3P	B3P
3l6g	B3P	B3P	B3P
3q4o	B3P	B3P	B3P

3q4o	B3P	B3P	B3P
2xsg	B3P	SIA	BTB
3lnl	B3P	PYR	IPH
3ib7	BTB	BTB	BTB
1y55	BTN	BTN	BTN
2c4i	BTN	BTN	BTN
4gda	BTN	BTN	BTN
1y55	BTN	BTN	BTN
2c4i	BTN	BTN	BTN
3ew2	BTN	ATP	AKG
4bj8	BTN	MLT	PEP
4z28	BTN	TAM	HED
2rde	C2E	C2E	C2E
4eu0	C2E	C2E	C2E
4foj	C2E	C2E	C2E
4urg	C2E	C2E	C2E
5euh	C2E	C2E	C2E
2f6u	CIT	CIT	CIT
3bhd	CIT	CIT	CIT
2r4i	CIT	CIT	CIT
3f7c	CIT	TAM	CIT
3gm5	CIT	P6G	CIT
3lmz	CIT	BTB	BTB
3nnb	CIT	MLT	MLT
3pko	CIT	ATP	TLA
3qsl	CIT	2GP	2GP
3rju	CIT	TAM	TAM
4hfv	CIT	GSH	PEP
4k7g	CIT	TAM	TAM
5kbf	CMP	ADP	ADP
